# Adrenal Gland Microenvironment and Its Involvement in the Regulation of Stress-Induced Hormone Secretion during Sepsis

**DOI:** 10.3389/fendo.2016.00156

**Published:** 2016-12-14

**Authors:** Waldemar Kanczkowski, Mariko Sue, Stefan R. Bornstein

**Affiliations:** ^1^Department of Internal Medicine III, Technische Universität Dresden, Dresden, Germany; ^2^Department of Endocrinology and Diabetes, King’s College London, London, UK

**Keywords:** stress system, hypothalamic–pituitary–adrenal axis, immune–adrenal crosstalk, ACTH, glucocorticoids

## Abstract

Survival of all living organisms depends on maintenance of a steady state of homeostasis, which process relies on its ability to react and adapt to various physical and emotional threats. The defense against stress is executed by the hypothalamic–pituitary–adrenal axis and the sympathetic–adrenal medullary system. Adrenal gland is a major effector organ of stress system. During stress, adrenal gland rapidly responds with increased secretion of glucocorticoids (GCs) and catecholamines into circulation, which hormones, in turn, affect metabolism, to provide acutely energy, vasculature to increase blood pressure, and the immune system to prevent it from extensive activation. Sepsis resulting from microbial infections is a sustained and extreme example of stress situation. In many critical ill patients, levels of both corticotropin-releasing hormone and adrenocorticotropin, the two major regulators of adrenal hormone production, are suppressed. Levels of GCs, however, remain normal or are elevated in these patients, suggesting a shift from central to local intra-adrenal regulation of adrenal stress response. Among many mechanisms potentially involved in this process, reduced GC metabolism and activation of intra-adrenal cellular systems composed of adrenocortical and adrenomedullary cells, endothelial cells, and resident and recruited immune cells play a key role. Hence, dysregulated function of any of these cells and cellular compartments can ultimately affect adrenal stress response. The purpose of this mini review is to highlight recent insights into our understanding of the adrenal gland microenvironment and its role in coordination of stress-induced hormone secretion.

## Introduction

In order to survive all, living organisms must maintain a steady state of homeostasis. This unique capacity to react and adapt to various physical and emotional threats in higher vertebrates is mediated by a coordinated action of the nervous, endocrine, and immune systems, known as stress system (1). Activation of the stress system, which is composed of the hypothalamic–pituitary–adrenal (HPA) axis and the sympathetic–adrenal medullary system, leads to increased synthesis and release of glucocorticoids (GCs) and catecholamines (CAs) from the adrenal cortex and medulla, respectively (2–4). The main function of these hormones is maintenance and restoration of basal and stress-related body homeostasis. This protective action of adrenal hormones is mostly accomplished by their metabolic, cardioprotective, and anti-inflammatory actions. In particular, both GCs and CAs are known to acutely enhance plasma glucose levels and promote an increased cardiac output and high blood pressure, while protecting against excessive inflammation (5–8).

Secretion of adrenal hormones during stress-free conditions is regulated centrally and is characterized by ultradian rhythms (9). An early morning release of adrenocorticotropic hormone (ACTH) into circulation is enhanced by increased concentrations of corticotropin-releasing hormone (CRH) and arginine vasopressin (AVP) in the hypophyseal portal system of anterior pituitary (10, 11). Consequently, elevated plasma levels of ACTH stimulate adrenocortical cells to produce and release GC hormones. In turn, as a part of negative feedback, GCs act directly on the pituitary gland to reduce ACTH secretion and on hypothalamic neurons to reduce CRH release (11, 12). Systemic rise in CA levels is in turn initiated by activation of the sympathetic nervous system and splenic nerves innervating the adrenal medulla (13, 14).

**Figure 1:**
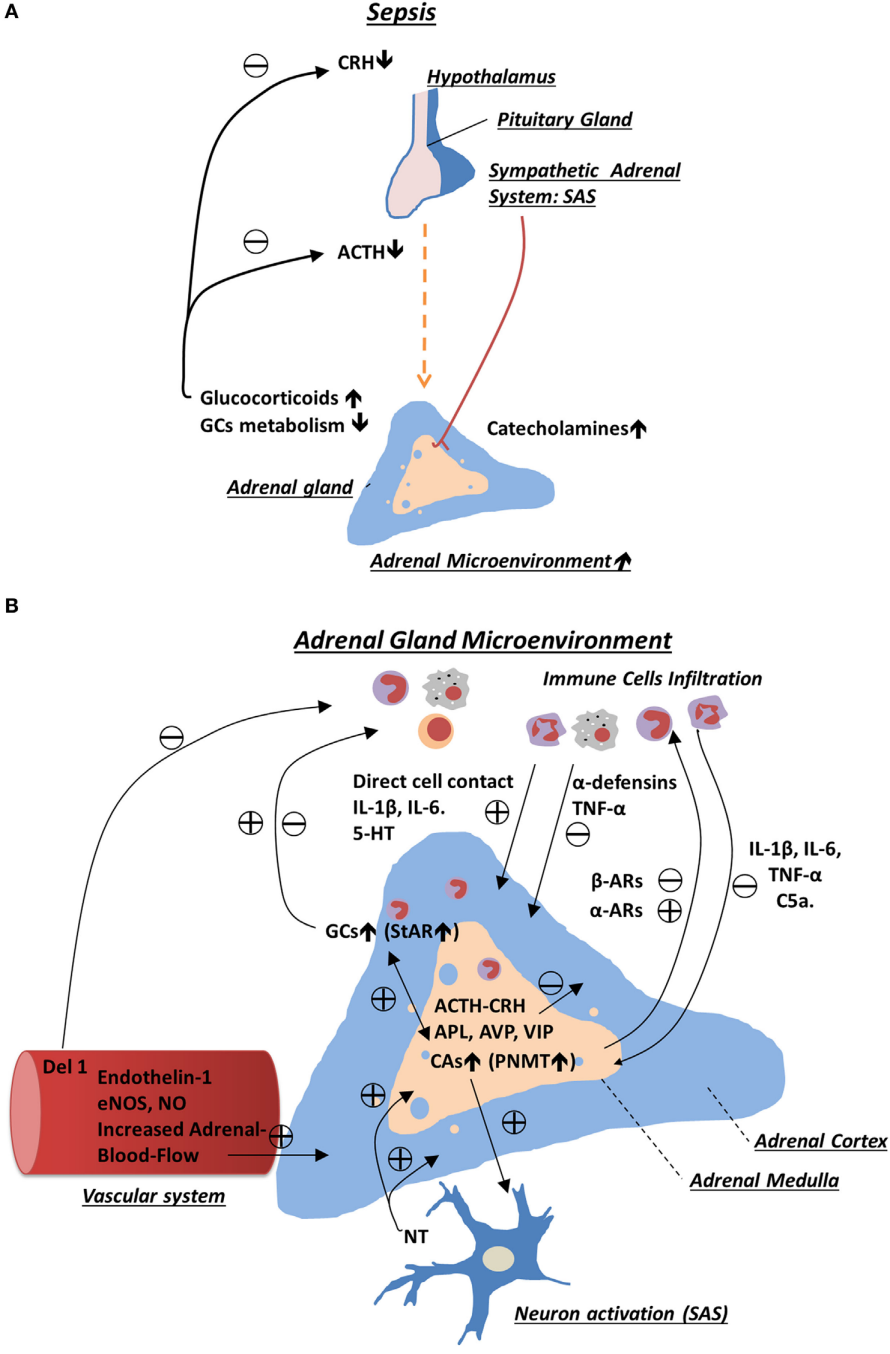
**Schematic representation of regulatory mechanisms involved in adrenal gland stress response during sepsis**. **(A)** Sepsis activates the hypothalamic–pituitary–adrenal axis and sympathetic–adrenal medullary system, which in turn enhances production of glucocorticoids (GCs) and catecholamines from the adrenal gland. During chronic phase of sepsis, production of corticotropin-releasing hormone and adrenocorticotropic hormone (ACTH) is inhibited as a result of increased negative feedback from the elevated levels of GCs. High level of adrenal GCs in plasma is sustained due to stimulatory effects provided by intra-adrenal microenvironment and reduced GCs metabolism. **(B)** In the adrenal gland, many cell types including resident and infiltrating immune cells, sympathetic neurons, endothelial cells, and adrenal chromaffin and adrenocortical cells interact with each other. This interaction occurs through a direct cell–cell contact and in a paracrine way, which ultimately leads to sustained production of adrenal hormones. Among many mediators produced in adrenal gland, which are reported to modulate adrenal hormone production, are adrenocortical steroids, catecholemines, and various cytokines such as interleukin (IL)-1β, IL-6, tumor necrosis factor-α, serotonin (5-HT), neurotransmitters, and neurohormones (e.g., APL, ACTH, and vasoactive intestinal peptide).

One of the extreme examples of sustained and severe physical stress is sepsis syndrome. It is characterized by abnormal host response to infection, resulting in systemic inflammation that frequently culminates in a life-threatening dysfunction of multiple organs (15, 16). Severe sepsis remains the leading cause of mortality worldwide, and its incidence is increasing (17, 18). An intact function of the stress system, and in particular the activation of GC and CA production, is critical to survive this adverse condition (19, 20). During sepsis, this homeostatic function of the stress system is frequently impaired with mechanisms remaining largely unknown (21, 22). Consequently, many critically ill patients demonstrate suppressed ACTH and CRH levels while having normal or elevated cortisol levels (22). Hence, a key involvement of pituitary-independent factors was proposed (2). Among these, reduced GC metabolism and activation of local adrenal microenvironment seem to play crucial role (23, 24) (Figure 1).

As regulation of the stress system and importance of impaired metabolism of GCs during sepsis were recently presented and discussed in many review articles (25–27), the main purpose of this mini review is to highlight recent insights into our understanding of the role local adrenal gland microenvironment in the regulation of stress-induced hormone secretion during sepsis.

## Adrenal Gland Microenvironment

Within adrenal gland, two embryonically different tissues coexist: mesodermally derived, steroid-producing cortex and ectodermally derived, CA-producing medulla. Within these two environmental niches, interplay between various cells takes place including adrenocortical and chromaffin cells, neuronal cells, immune cells, endothelial cells, and glia cells (28). The adrenal gland is a source of many bioactive substances including steroid hormones, CAs, cytokines, neurotransmitters, and neuropeptides (Table 1). These substances are known to interact with various cell types within adrenal gland itself, thereby influencing its function during stress conditions and disease (24, 28).

**Table 1:** **Intra-adrenal interaction involved in a regulation of adrenal stress response**.

Cell interactions	Mediators	Action
**Immune–adrenal crosstalk** (macrophages, neutrophils, mast cells, lymphocytes)	Glucocorticoids (GCs)	Stimulatory effects on naïve and inhibitory actions on activated immune cells
	Cytokines	Stimulatory [interleukin (IL)-1β, IL6] or inhibitory action [tumor necrosis factor (TNF)-α] on adrenal steroidogenesis
	Catecholamine (CA)	Anti-inflammatory effects through activation of β-adrenergic receptors (ARs) (increase IL-10, decrease TNF-α in macrophage, and decrease NO and ROS in neutrophils)
		Pro-inflammatory effects through activation of α-ARs
	Direct contact	Stimulatory effects on adrenal DHEA synthesis
	α-Defensins	Inhibitory effects on steroidogenesis through decreased sensitivity of adrenal cells to adrenocorticotropic hormone (ACTH)
	Neutrophil extracellular traps	Destruction of adrenal cells and promotion of hemorrhages

**Adrenocortical–adrenomedullary interactions** (adrenocortical cells and chromaffin cells)	GCs	Stimulatory action on CA synthesis (through PNMT upregulation)
	CAs	Stimulatory action on adrenal steroidogenesis
	Neurotransmitters (NT) (substance P, vasoactive intestinal peptide, neuropeptide Y, etc.)	Stimulatory and inhibitory action on adrenal steroidogenesis
	Local corticotropin-releasing hormone–ACTH system	Stimulatory action on adrenal steroidogenesis
	Apelinergic system	Stimulatory action on adrenal steroidogenesis

**Vasculature** (endothelial cells)	Endothelin-1	Direct stimulatory effect on GC production
	eNOS and NO	Possible stimulatory effect on steroidogenesis
	Developmental endothelial locus 1	Regulation of immune cells recruitment into adrenal gland
	Adrenal blood flow	Stimulatory effect on steroidogenesis

**Innervation** (sympathetic neurons)	NT (acetylcholine)	Increased steroidogenesis *via* enhanced sensitivity of adrenal cells to ACTH
		Increased production and secretion of CAs

### Adrenal Cortex and Medulla Interactions during Sepsis

Ample evidence exists suggesting a bidirectional interaction between adrenal cortex and the medulla (28). Despite a classical view on the adrenal gland anatomy, demonstrating a clear separation of the cortex and medulla, it has been well documented that these two endocrine tissues are morphologically intermingled (29, 30). This close localization, in turn, enables direct cell–cell and paracrine interactions between adrenocortical and chromaffin cells involving their secretory products.

Consequently, the adrenal cortex-derived GCs were found to enhance synthesis of CAs both *in vitro* (31) and *in vivo* (32–34). GCs were found to execute these effects by upregulating expression of phenylethanolamine *N*-methyltransferase (PNMT), which gene encodes for an enzyme that catalyzes the synthesis of epinephrine from norepinephrine. Furthermore, studies using various knockout mice, which were deficient in steroidogenic factor-1, glucocorticoid receptor, or corticotropin-releasing hormone type 1 receptor, clearly demonstrated that lack of one of the these key steroidogenic regulators impair PNMT gene expression and thus basal and stress-induced epinephrine production (35–38). Similarly, an intact function of adrenal medulla is important to maintain adrenocortical function. It has been shown that coculture of bovine adrenocortical cells with chromaffin cells enhanced a basal GC secretion by 10-fold (39). Furthermore, CAs were found to enhance production of various steroids including cortisol, aldosterone, and androstenedione, through mechanism involving upregulation of steroidogenic acute regulatory protein (28, 40, 41).

During sepsis, an enhanced production of epinephrine and norepinephrine (42–44) and increased secretory capacity of adrenal chromaffin cells were reported (45). Unlike expression of tyrosine hydroxylase, which enzyme is involved in first step of CA biosynthesis, upregulation of PNMT gene expression in adrenal medulla was found to be regulated independently of splanchnic nerve stimulation (28). Instead, an extremely high concentration of GCs was reported to be involved in regulation of PNMT expression during sepsis (46). Consequently, stress-induced expression of PNMT is strongly attenuated in CRH-deficient mice (47).

In addition, adrenal chromaffin cells release many active neuropeptides and transmitters, including neuropeptide Y (NPY), vasoactive intestinal peptide (VIP), or substance P, with known stimulatory action on GCs production (48, 49). For example, VIP, which levels increase during experimental endotoxemia in adrenal medulla, was found to stimulate DHEA, testosterone, androstenedione, cortisol, and aldosterone secretion in adrenocortical cells (48–52). Among other mediators found in adrenal medulla, which may influence GC production during sepsis, are angiotensin, AVP, CRH, ACTH, and apelin (53–55). Although direct stimulatory effects of angiotensin 2, CRH, and ACTH on adrenal hormone production are well described, the degree to which each of these peptides contributes to hormone production during sepsis conditions has not been fully elucidated yet (2, 56). Another interesting peptide found recently to be expressed in the adrenal medulla is apelin. Apelin is a neurohormone, which acts through its receptor APJ expressed in various organs including hypothalamic neurons, anterior pituitary, and adrenal gland. It was recently found to modulate neuroendocrine response to stress through stimulation of secretion of both ACTH and corticosterone directly and *via* AVP- and CRH-dependent pathways (54, 55, 57).

Recently, a population of nestin-positive glia-like multipotent stem cells was identified in adrenal medulla of nestin-GFP transgenic mice (58). Interestingly, when subjected to chronic stress, this population was able to give rise to new chromaffin cells, suggesting a direct involvement of these cells in the adrenal stress adaptation (58–60). It is therefore very interesting to investigate the fate of this cells and their potential interaction with adrenocortical cells during sepsis conditions.

In summary, an intact function of both adrenal cortex and adrenal medulla is of pivotal importance in the regulation of adrenal stress response. Consequently, any disorders or medications that can attenuate adrenal cortex function will also ultimately affect CAs production by chromaffin cells and *vice versa* potentially impacting the outcome of many stress-related disorders (19).

### Immune–Adrenal Crosstalk

In the last 40 years, it has become evident that immune and endocrine systems interact with each other at the level of adrenal gland and that this interaction is crucially involved in the regulation of adrenal gland function during normal and stress conditions (61). This immune–endocrine crosstalk is mostly executed by bidirectional action of paracrine factors, such as steroid hormones, CAs, and various vasoactive or proteolytic enzymes, as well as by direct cell–cell contacts and activation of toll-like receptors (TLRs) (28, 62).

#### Immune–Adrenal Interaction at the Level of Adrenal Cortex

During non-stress conditions, many immune cells can be found in the innermost zone of adrenal cortex known as *zona reticularis*; these are particularly tissue macrophages (63), dendritic cells (64), mast cells (65), and lymphocytes (66). However, some of them, especially macrophages and mast cells, were also reported in other parts of the adrenal gland including subcapsular region and in the adrenal medulla (64). In non-stress conditions, adrenal-resident immune cells play important homeostatic functions by sensing pathogens, removing apoptotic cells, and promoting tissue remodeling through, e.g., secretion of growth factors (24, 67). Recently, a new population of adrenal macrophages was identified based on the high expression of a complement receptor immunoglobulin, CRIg, which molecule is known to mediate phagocytosis of pathogens and apoptotic cells (68). During systemic inflammation induced either by LPS injection or CLP, highly dynamic changes within adrenal immune cell populations can be observed. For instance, it has been shown that already within first 3 h after administration of LPS or induction of peritonitis in rodents, a rapid infiltration of neutrophils takes place, while the number of local dendritic cells and macrophages declines (69–71).

Using electron microscopy, close cell–cell localization between local immune cells and surrounding adrenocortical, chromaffin, or endothelial cells was demonstrated (64, 66). This in turn enables various bidirectional and direct interactions between each cell type. For example, a functional crosstalk between adrenocortical cells, expressing major histocompatibility complex class II molecules (MHC/HLA), and leukocytes was described. Indeed, a co-incubation of T cells with primary cultures of human adrenocortical cells promoted adrenal androgen and cortisol secretion (66). Besides direct interaction, various immune cells were found to regulate adrenal hormone production in a paracrine way through secretion of cytokines, such as interleukins (IL) 1 and 6 (72), and pro-opiomelanocortin (POMC)-derived peptides, e.g., ACTH and biogenic amines (28, 73).

The ample experimental evidence exists demonstrating that cytokines are critically involved in ACTH-independent activation of GC production during sepsis (72). For example, injection of IL-1β enhanced GC production in hypophysectomized rats (74). Furthermore, an impaired synthesis of GCs after LPS injection was observed in mice receiving sera against tumor necrosis factor (TNF)-α and IL-6 and, to a lesser extent, IL-1β- or in IL-6-deficient mice (75, 76). However, it has been demonstrated that cytokine levels found normally in plasma are far too low to induce hormone production from adrenocortical cells. Therefore, the main source of cytokines must come from the adrenal gland itself (28). Sustained production of cytokines in highly anti-inflammatory environment of the adrenal gland during systemic inflammation was found to be enabled by increased expression of migration inhibitory factor (77). During sepsis, various cell types present within adrenal gland microenvironment may be a potential source of pro-inflammatory cytokines. For example, adrenocortical cells were found to express several TLRs and secrete several cytokines in response to bacterial ligands (78, 79). However, in a recent study, inactivation of myeloid but not adrenocortical TLR signaling resulted in significant reduction of intra-adrenal cytokine levels and activation of the HPA axis after LPS administration (80). The latter observation suggests that immune cells are the key sources of cytokines in the adrenal gland during sepsis. Due to high expression of both cytokine and GC receptors in adrenocortical and immune cells, respectively, cytokines and adrenal hormones are known to regulate production in each other. For example, IL-1β or IL-6 was found to increase adrenal hormone production, whereas TNF stimulation demonstrated rather opposite effect (81–83). In turn, adrenal steroid hormones are known to influence immune cells function. In particular, it has been demonstrated that in naïve immune cells, GCs can activate several inflammatory-related genes, e.g., TLRs, yet in cells treated with LPS, they inhibit inflammation (6). In a recent study, a chronic exposure to GCs was found to cause a shift in the innate–adaptive balance of the immune response, particularly, influencing the chemokine–chemokine receptor networks (84). Besides cytokines, other biologically active substances, such as serotonin (5-HT) or histamine, which are stored by mast cells show stimulatory effects on adrenal steroidogenesis (85, 86).

During prolonged sepsis, the immune–adrenal interaction may also result in the suppression of adrenal hormone production. Despite their important role in host defenses against bacterial infections limiting bacterial spread in circulation (87), a prolonged exposure of adrenal cells to neutrophil-derived antimicrobial agents, such as ROS and proteolytic enzymes secreted during formation of neutrophil extracellular traps, results in tissue damage (88). In addition, neutrophils were found to secrete corticostatins, e.g., α-defensins, which substances are known to interfere with ACTH-mediated increase in adrenocortical hormone production (89). In a recent study using developmental endothelial locus 1 (Del-1)-deficient mice (90), an association between higher amount of infiltrating neutrophils and impaired adrenal corticosterone production after systemic LPS administration was found (91).

#### Immune–Adrenal Interaction at the Level of Adrenal Medulla

Activation of the sympathetic nerve system during sepsis results in enhanced production and secretion of CAs from the adrenal chromaffin cells (44) through a mechanism involving an increased release of Ca^2+^ from endothelial reticulum (45). An early increase in endogenous CA level plays an important protective role as its absence due to either pharmacological or surgical intervention resulted in induction of hypotension and increased mortality of rats during experimental endotoxemia (20, 43). One of the mechanisms involved in the protective role of CAs, besides well-known vasopressor function, is the anti-inflammatory action on various immune cells. The immunomodulatory effects of CAs are mediated *via* their direct interactions with β-adrenergic receptors (ARs) expressed by a variety of immune cells. In particular, it has been shown that CAs promote IL-10 secretion while decreasing production of pro-inflammatory TNF cytokine in LPS-treated macrophages (92). Furthermore, incubation of neutrophils with CAs decreased NO production and ROS generation (93). However, these immune-suppressive effects of CAs should be carefully interpreted as they may correspond predominantly to the leukocyte populations or be restricted to β-AR activation. In fact, recently both epinephrine and norepinephrine were shown to increase IL-6 production in endothelial cell line: HMEC-1 and in human skin microvascular endothelial cells (94). It has been also found that activation of α-ARs in macrophages by CA may potentiate inflammation by increasing TNF-α production (95). Recently, phagocytes were shown to be able not only to secrete CAs in response to LPS stimulation but also to possess all major CA-generating and -degrading enzymes required for their production and inactivation (96). This discovery adds additional complexity to already complex immune–chromaffin cell interaction. During sepsis, the adrenal medulla shows high degree of immune infiltration (69). This suggests that overactivation of immune cells could potentially impair production and secretion of adrenomedullary hormones or induce structural damage. Indeed, during experimental sepsis induced by CLP, systemic inflammation resulted in a strong apoptosis in the adrenal chromaffin cells, which process required activation of receptors for complement C5a (97). The latter observation suggests that during progression of sepsis, an increased activation of immune system promoting apoptosis of chromaffin cells may impact stress-induced CA production.

### Adrenal Vasculature

Vascular system plays a key role in the proper functioning of many vital organs during sepsis. Besides enabling the secretion of steroid hormones and CAs into circulation, adrenal vasculature was found to control leukocyte infiltration through expression of adhesion molecules and Del-1 protein (91).

Adrenal cortex is characterized by high density of endothelial cells that stay in close vicinity with steroid-producing cells. Consequently, both adrenal hormones and endothelial cell-derived products were shown to influence function of adrenal vasculature and steroid-producing cells, respectively. For example, human adrenocortical cells increase production of aldosterone and cortisol once incubated with endothelial cell-conditioned media. Although the exact composition of the latter media was not determined, this stimulatory effect was found to require cAMP, but not PKA, pathway (98). Endothelial cells are known to produce several factors that, e.g., nitric oxide (NO) or endothelin-1, could potentially influence adrenal hormone production. In particular, in rat and human adrenocortical cells, it has been found that endothelin-1 can directly promote GC synthesis and potentiate angiotensin II and ACTH-induced aldosterone production (99). Besides endothelin-1, nitric oxide (NO) was found to exert stimulatory effect on adrenal steroidogenesis by mediating the acute response to ACTH (28). In fact, blocking of NO production either by using NO synthase inhibitor or NO scavengers resulted in decreased ACTH-mediated corticosterone release (100). However, in other studies using perfused adrenal glands administration of l-arginine, the substrate for nitric oxide synthesis did not change corticosterone response to ACTH, suggesting an indirect modulatory action of NO (101).

During sepsis, in many critically ill patients, an increased size of adrenal gland resulting from the hypervascularization and increased blood flow and adrenal hyperplasia were found to positively correlate with survival rate (102). Although an intact activity of splanchnic nerve is crucial in regulation of adrenal blood flow, other factors such as VIP, NPY, or neurotensin were found also to be involved (103, 104). Furthermore, a positive correlation between an increased blood flow and enhanced adrenal steroidogenesis was demonstrated (105). Indeed, an enhanced adrenal blood flow was found to participate in angiotensin II- and ACTH-induced aldosterone and cortisol production. This stimulatory action on adrenal vascular response to angiotensin II and ACTH was found to be mediated by vasoactive substances released from granules of mast cells (106). However, more recently, additional involvement of the cytochrome 450 in controlling the adrenal blood flow after AII and ACTH was demonstrated in rats (24, 107).

## Summary

Sepsis and septic shock strongly impacts body homeostasis, which if not sufficiently counteracted by activated stress system results in increased mortality of critically ill patients (56, 108, 109). During chronic phase of sepsis, elevated adrenal hormone secretion was reported to be mediated by pituitary-independent mechanisms including activation of intra-adrenal microenvironment (22, 23). An increasing amount of experimental studies support the key involvement of several cell types and cellular systems present within adrenal gland microenvironment in the sustained production of adrenal hormones during sepsis. Consequently, any impairment in function of these systems can ultimately affect adrenal stress response (2, 24).

## Author Contributions

WK, MS, and SB have all made substantial, direct, and intellectual contribution to the work and approved it for publication.

## Conflict of Interest Statement

The authors declare that the research was conducted in the absence of any commercial or financial relationships that could be construed as a potential conflict of interest. The reviewer OL and handling Editor declared their shared affiliation, and the handling Editor states that the process nevertheless met the standards of a fair and objective review.
